# Supplementary material for: Integrating temporal single-cell gene expression modalities for trajectory inference and disease prediction
Source: Genome Biol. 2022 Sep 5;23:186. doi: 10.1186/s13059-022-02749-0 (PMC9442962; doi:10.1186/s13059-022-02749-0)
Supplement: Supplementary file 1 — Additional file 1. Supplementary Tables S1-S3 and Supplementary Figures S1-17. [file 13059_2022_2749_MOESM1_ESM.pdf]

Supplementary Tables

Table S1: Datasets and Preprocessing Overview

ID	Description	Metadata	Task	Platform	Organism	Reference	Batch	Batch correction approach	Normalization
Nestorowa	hematopoiesis differentiation	FACS	TI	Smart-seq2	Mouse	mm10	NA	NA	Scran
Buettner	mouse embryonic cell cycle	FACS	TI	Smarter C1	Mouse	mm10	NA	NA	Scran
Engel	NKT cell differentiation	FACS	TI	Smart-seq2	Mouse	mm10	NA	NA	Scran
Olsson	hematopoiesis differentiation	FACS	TI	Smarter C1	Mouse	mm10	NA	NA	Scran
Lane	LPS stimulation	condition	classification	Smart-seq2	Mouse	mm10	library	ComBat concatenation	Scran with batch
Pollyea	AML chemotherapy	condition	classification	10X Genomics	Human	GRCh38	condition	ComBat concatenation	Scran with batch
Burkhardt	IFN-γ stimulation	condition	classification	10X Genomics	Human	GRCh38	patient	MNN concatenation	Scran with batch
Stetson	AML diagnosis/relapse	disease status	classification	Smart-seq2	Human	GRCh38	patient	ComBat concatenation	Scran with batch
Schafflick	MS case/control	disease status	classification	10X Genomics	Human	GRCh38	patient	ComBat concatenation	Scran with batch

Table S2: Overview of Optimized Parameters

Method name	Type	Parameter description	Parameters
Unintegrated	early	NA	NA
Concatentation	early	NA	NA
Sum	early	NA	NA
CellRank	early	weight given to velocity transition probability matrix<br>velocity similarity metric<br>velocity transition probability matrix mode	$\lambda \in$ [0.2, 0.8]<br>correlation, dot product, cosine<br>monte-carlo, dynamical
SNF	intermediate	number of nearest neighbors in affinity graph<br>kernel bandwidth parameter<br>number of eigenvectors	k = 5, 10, 25, 50<br>$\mu \in$ [0.4, 0.8]<br>K = 20, 50
Grassmann joint embedding	intermediate	number of nearest neighbors in affinity graph<br>kernel bandwidth parameter<br>tradeoff parameter between individual and merged subspaces<br>number of eigenvectors	k = 5, 10, 25, 50<br>$t \in$ [100, 500]<br>$\alpha \in$ [0.01, 2]<br>K = 20, 50
Integrated diffusion	intermediate	number of nearest neighbors in diffusion operator<br>number of clusters for denoising<br>number of eigenvectors	k = 5, 10, 25, 50<br>c = 5, 10, 25, 50<br>K = 20, 50
PRECISE	intermediate	number of principal vectors	npvs $\in$ [10, 50]
MOFA+	intermediate	NA	NA
Seurat v4	intermediate	NA	NA

Table S3: Cell Surface Markers Specified in Trajectory Inference Datasets

Hematopoietic Stem and Progenitor Cells from Nestorowa et al.		
name	ID	markers
long-term hematopoietic stem cells	LT-HSC	$\mathrm{Lin}^{-}\mathrm{c\text{-}Kit}^{+}\mathrm{Sca1}^{+}\mathrm{Flk2}^{-}\mathrm{CD34}^{-}$
lymphoid multipotent progenitors	LMPP	$\mathrm{Lin}^{-}\mathrm{c\text{-}Kit}^{+}\mathrm{Sca1}^{+}\mathrm{Flk2}^{+}\mathrm{CD34}^{+}$
multipotent progenitors	MPP	$\mathrm{Lin}^{-}\mathrm{c\text{-}Kit}^{+}\mathrm{Sca1}^{+}\mathrm{Flk2}^{-}\mathrm{CD34}^{+}$
megakaryocyte-erythrocyte progenitors	MEP	$\mathrm{Lin}^{-}\mathrm{c\text{-}Kit}^{+}\mathrm{Sca1}^{-}\mathrm{CD16/32}^{-}\mathrm{CD34}^{-}$
common myeloid progenitors	CMP	$\mathrm{Lin}^{-}\mathrm{c\text{-}Kit}^{+}\mathrm{Sca1}^{-}\mathrm{CD16/32}^{-}\mathrm{CD34}^{+}$
granulocyte-monocyte progenitors	GMP	$\mathrm{Lin}^{-}\mathrm{c\text{-}Kit}^{+}\mathrm{Sca1}^{-}\mathrm{CD16/32}^{+}\mathrm{CD34}^{+}$
**Natural Killer T Cells from Engel et al.**		
name	ID	markers
natural killer T cell 0	NKT0	$\mathrm{CD8}\alpha^{-}\mathrm{CD1d\text{-}}\alpha\mathrm{\text{-}GalCer}\ \mathrm{TCR}\beta^{\mathrm{int}}\mathrm{CD24}^{\mathrm{hi}}\mathrm{CD44}^{\mathrm{lo}}\mathrm{NK1.1}^{-}\mathrm{v}\alpha 14$
natural killer T cell 1	NKT1	$\mathrm{CD24}^{\mathrm{lo}}\mathrm{TCR}\beta^{\mathrm{lo}}\mathrm{NK1.1}^{\mathrm{hi}}\mathrm{CD27}^{\mathrm{hi}}\mathrm{CCR6}^{-}\mathrm{v}\alpha 14$
natural killer T cell 2	NKT2	$\mathrm{CD24}^{\mathrm{lo}}\mathrm{TCR}\beta^{\mathrm{hi}}\mathrm{NK1.1}^{-}\mathrm{CD27}^{\mathrm{hi}}\mathrm{CD4}^{\mathrm{hi}}\mathrm{v}\alpha 14$
natural killer T cell 17	NKT17	$\mathrm{CD24}^{\mathrm{lo}}\mathrm{CD27}^{\mathrm{lo}}\mathrm{CD4}^{-}\mathrm{CCR6}^{+}\mathrm{CD103}^{\mathrm{hi}}\mathrm{v}\alpha 14$
**Hematopoietic Progenitor Cells from Olsson et al.**		
name	ID	markers
lineage negative	LSK	$\mathrm{Lin}^{-}\mathrm{Sca1}^{+}\mathrm{CD117}^{+}$
common myeloid progenitor	CMP	$\mathrm{Lin}^{-}\mathrm{CD117}^{+}\mathrm{Sca1}^{-}\mathrm{CD16/32}^{\mathrm{dim}}\mathrm{CD34}^{+}$
granulocyte monocyte progenitor	GMP	$\mathrm{Lin}^{-}\mathrm{CD117}^{+}\mathrm{Sca1}^{-}\mathrm{CD16/32}^{+}\mathrm{CD34}^{+}$

Supplementary Figures

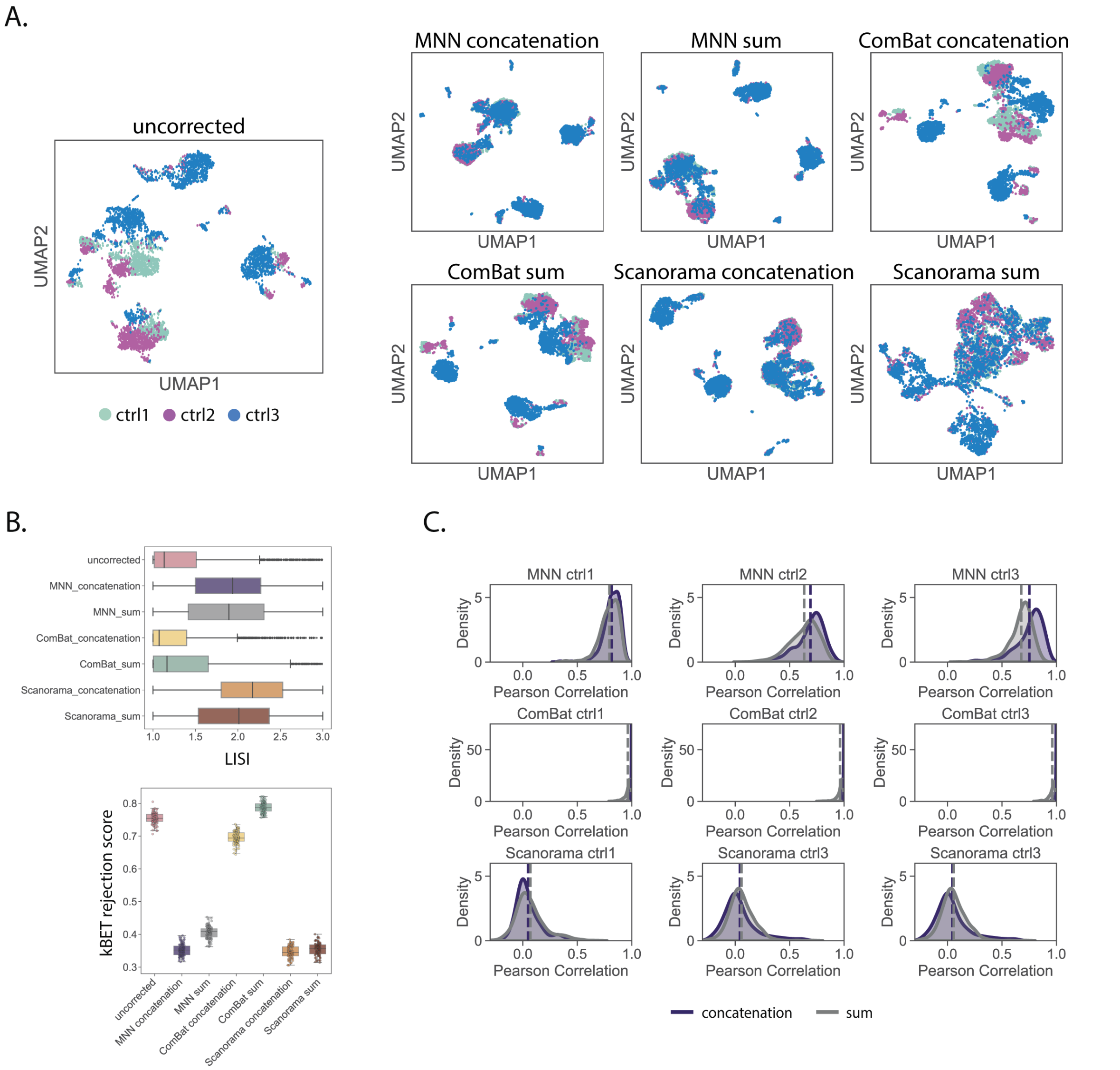

Figure S1: Evaluating batch effect correction for control pancreatic islet cells in INFγ stimulation dataset. (A) UMAP visualization of control pancreatic islet cells across batch correction strategies. Spliced and unspliced modalities were combined via concatenation or sum prior to correction with mutual nearest neighbors (MNN), ComBat, or Scanorama. Method performance was measured by batch label mixing metrics kBET and LISI (B), as well as the preservation of the relationship between spliced and unspliced counts (C). Distributions represent the per gene Pearson correlation between cell-cell distances in the phase space (unspliced, spliced) of corrected data and the cell-cell distances in the phase space of each individual donor. Dashed line represents the mean correlation.

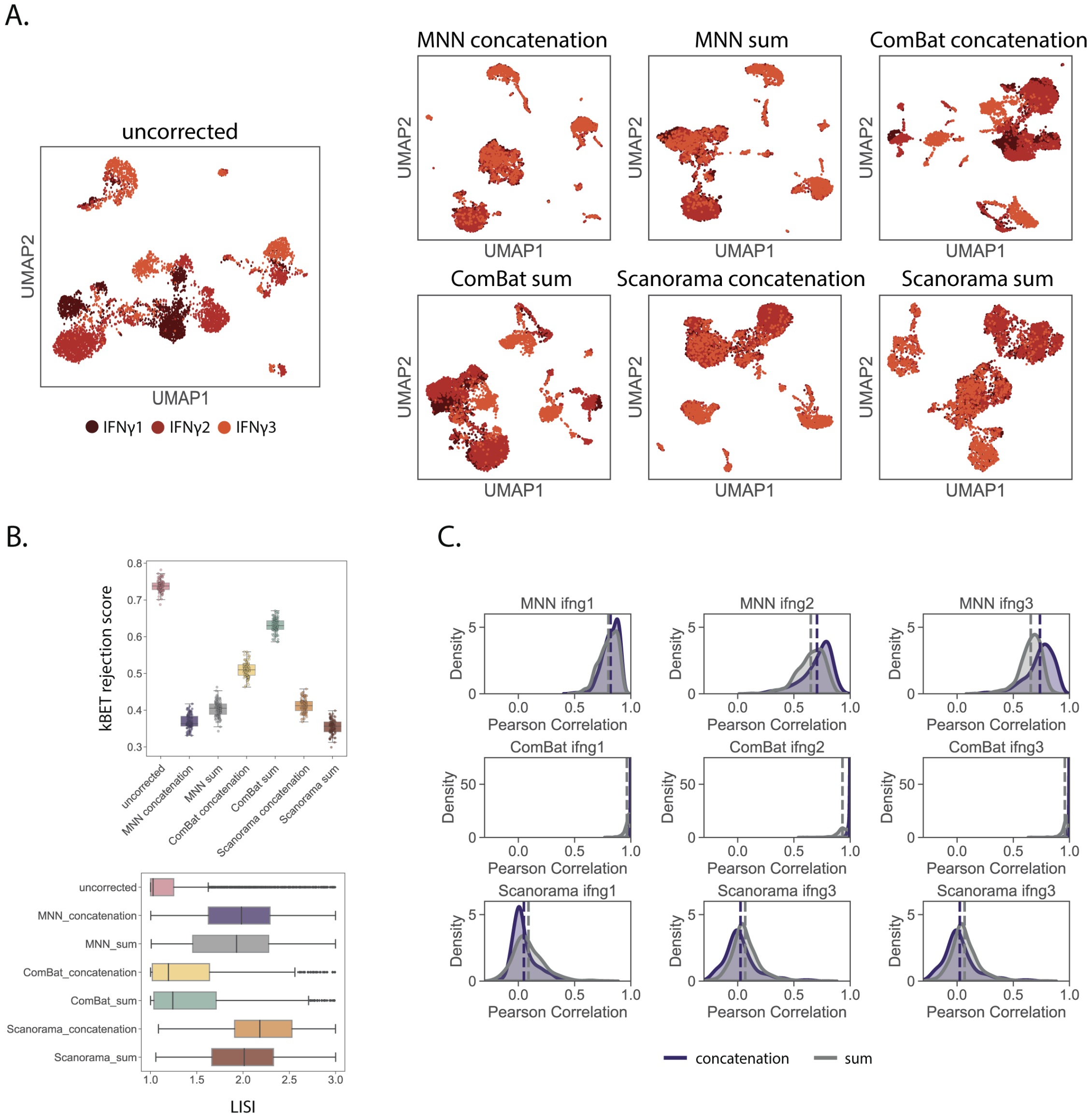

Figure S2: Evaluating batch effect correction for INFγ stimulated pancreatic islet cells in INFγ stimulation dataset. (A) UMAP visualization of INFγ stimulated pancreatic islet cells across batch correction strategies. Spliced and unspliced modalities were combined via concatenation or sum prior to correction with mutual nearest neighbors (MNN), ComBat, or Scanorama. Method performance was measured by batch label mixing metrics kBET and LISI (B), as well as the preservation of the relationship between spliced and unspliced counts (C). Distributions represent the per gene Pearson correlation between cell-cell distances in the phase space (unspliced, spliced) of corrected data and the cell-cell distances in the phase space of each individual donor. Dashed line represents the mean correlation.

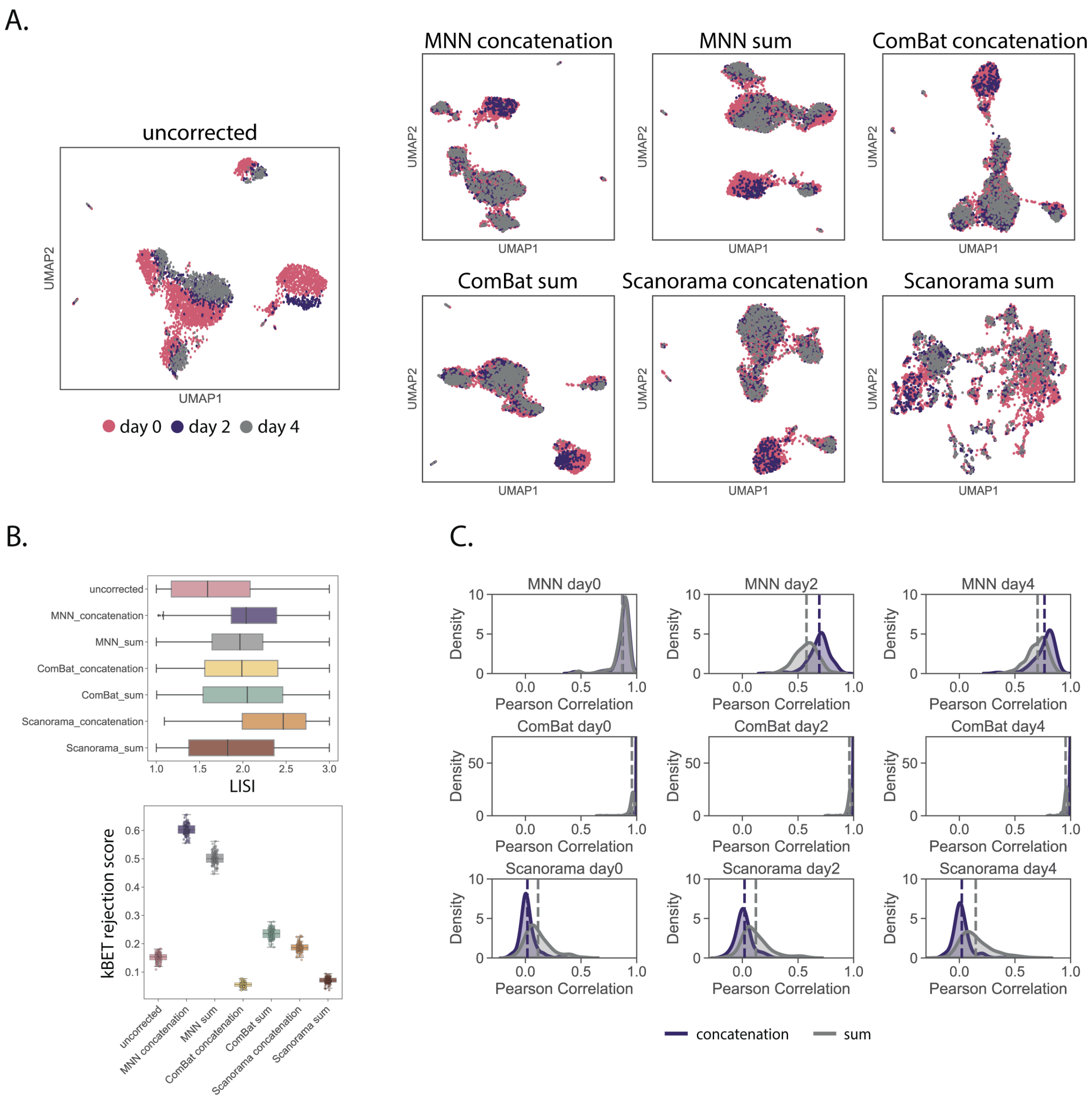

Figure S3: Evaluating batch effect correction for AML chemotherapy treated cells. (A) UMAP visualization of AML chemotherapy treated cells across batch correction strategies. Spliced and unspliced modalities were combined via concatenation or sum prior to correction with mutual nearest neighbors (MNN), ComBat, or Scanorama. Method performance was measured by batch label mixing metrics kBET and LISI (B), as well as the preservation of the relationship between spliced and unspliced counts (C). Distributions represent the per gene Pearson correlation between cell-cell distances in the phase space (unspliced, spliced) of corrected data and the cell-cell distances in the phase space of each time point (day 0, day 2, day 4). Dashed line represents the mean correlation.

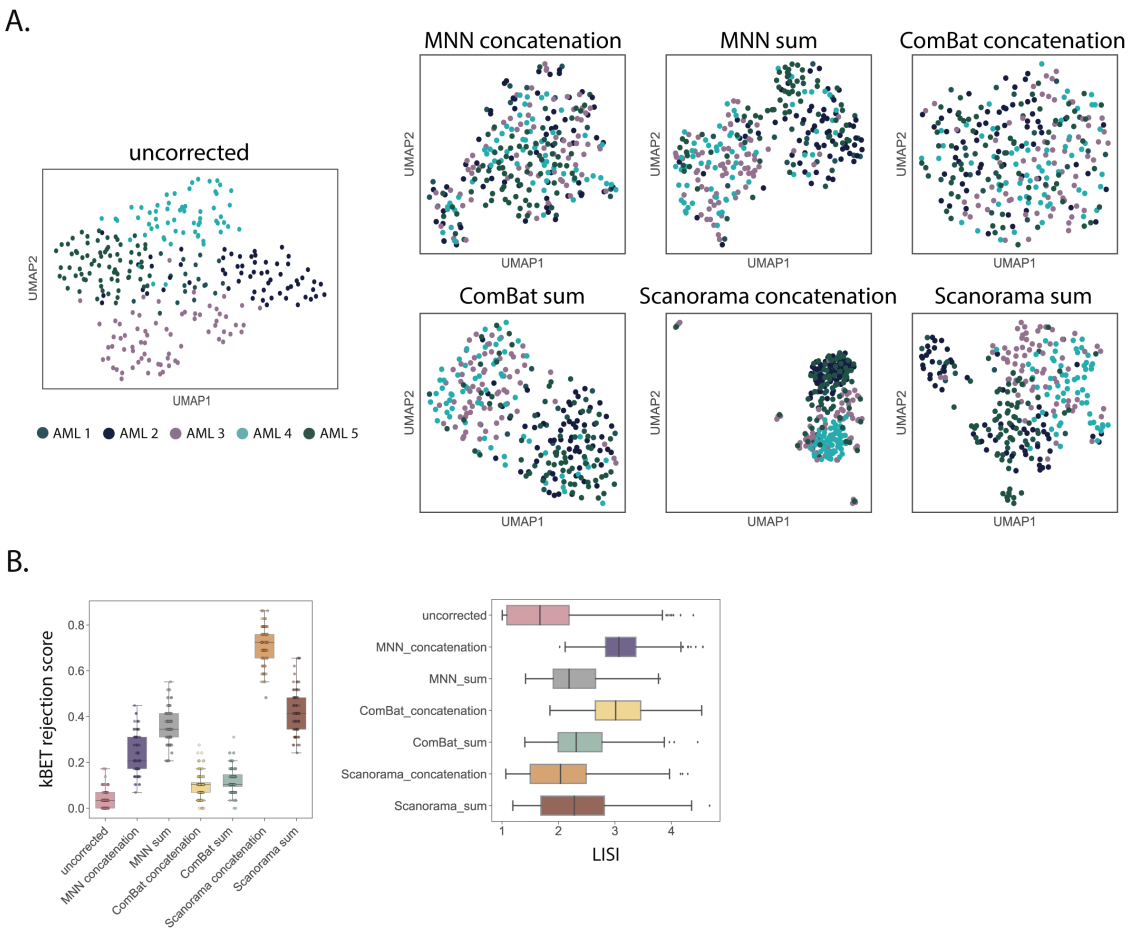

Figure S4: Evaluating batch effect correction for AML diagnosis patient cells in AML diagnosis/relapse dataset. (A) UMAP visualization of AML diagnosis patient cells across batch correction strategies. Spliced and unspliced modalities were combined via concatenation or sum prior to correction with mutual nearest neighbors (MNN), ComBat, or Scanorama. (B) Method performance was measured by batch label mixing metrics kBET and LISI across patients.

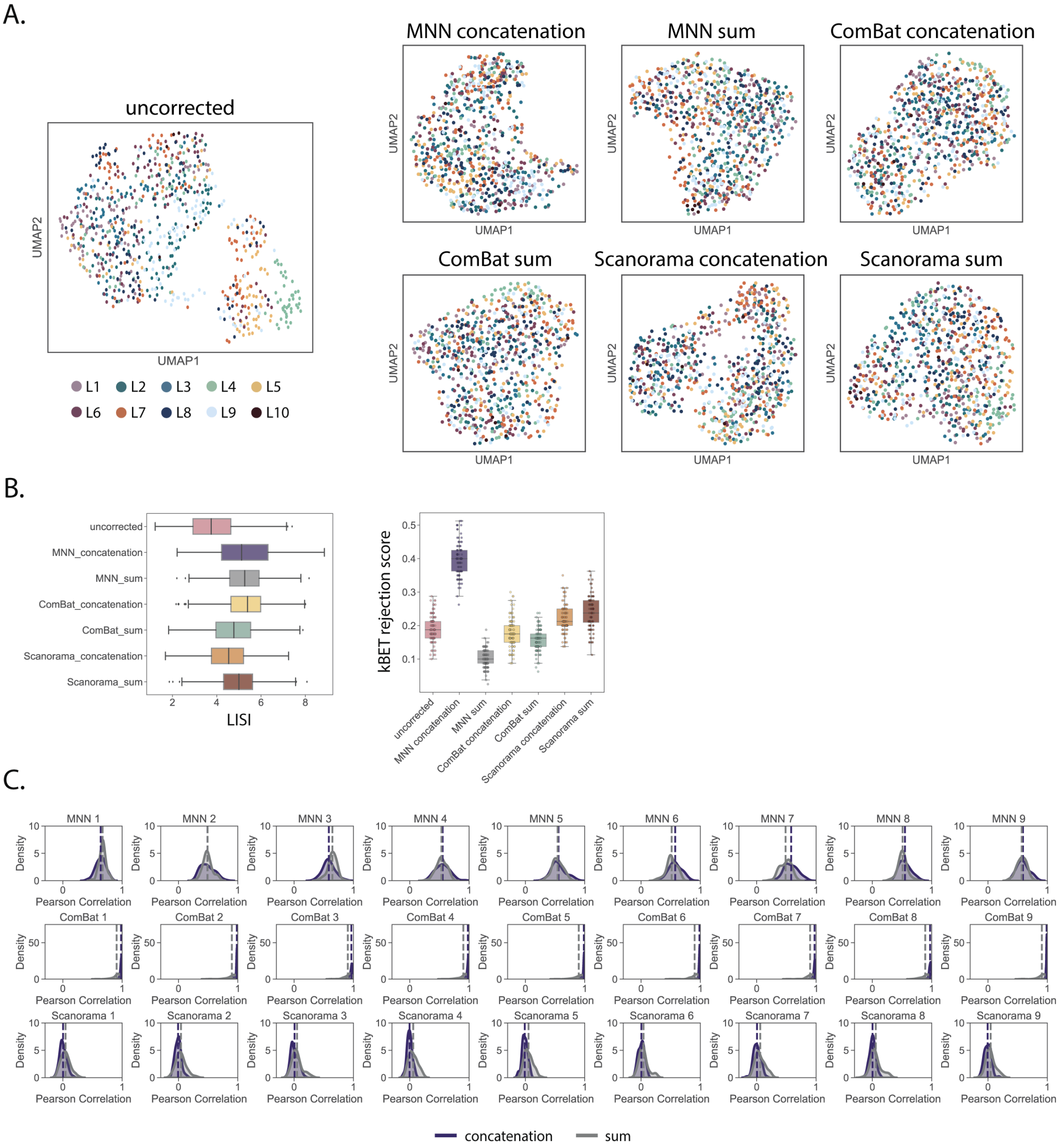

Figure S5: Evaluating batch effect correction for LPS stimulated cells. (A) UMAP visualization of LPS stimulated cells across batch correction strategies. Spliced and unspliced modalities were combined via concatenation or sum prior to correction with mutual nearest neighbors (MNN), ComBat, or Scanorama. Method performance was measured by batch label mixing metrics kBET and LISI (B), as well as the preservation of the relationship between spliced and unspliced counts (C). Distributions represent the per gene Pearson correlation between cell-cell distances in the phase space (unspliced, spliced) of corrected data and the cell-cell distances in the phase space of each individual library. Dashed line represents the mean correlation. Library 10 was excluded from correlation analysis as it contained too few cells.

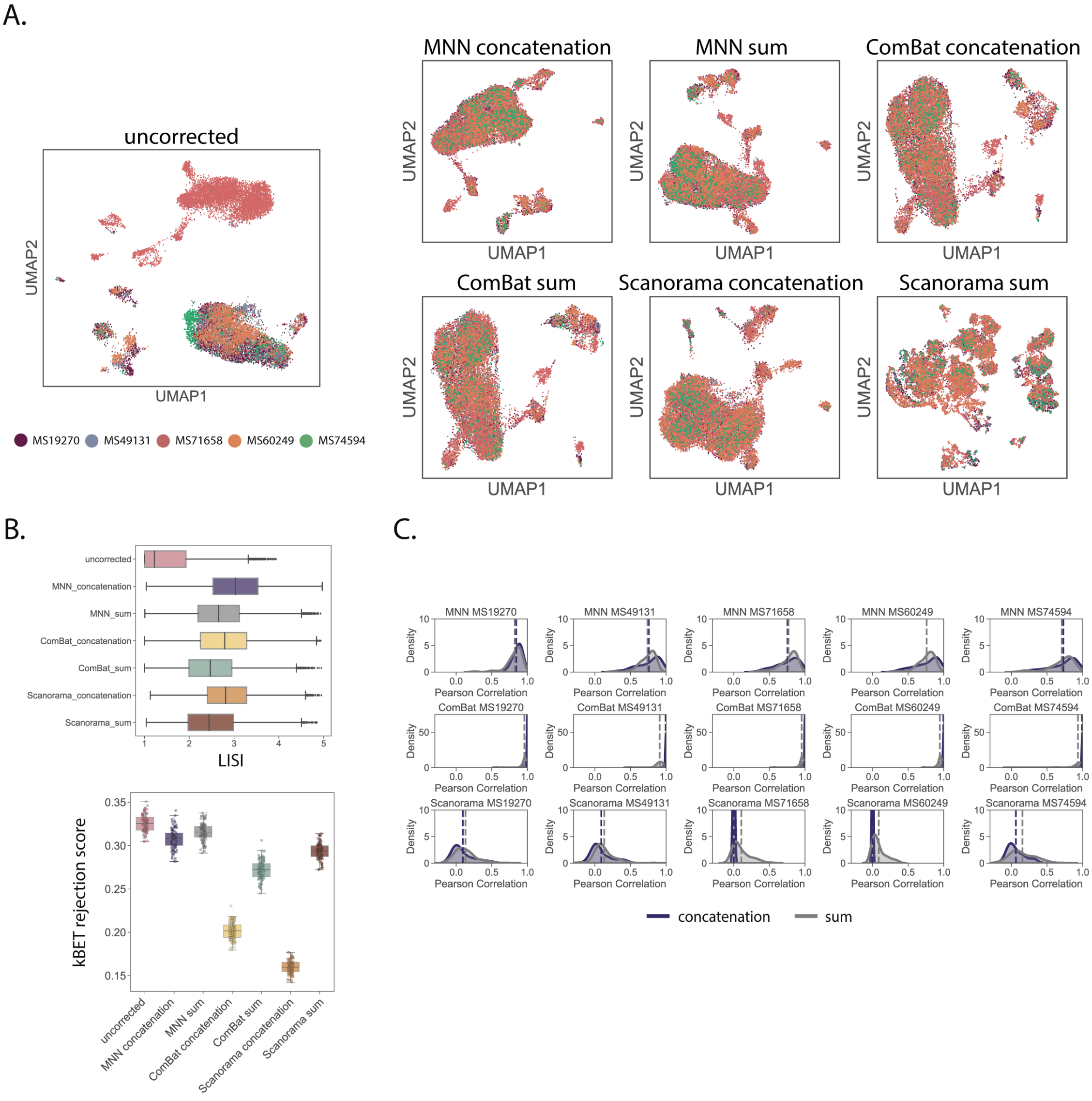

Figure S6: Evaluating batch effect correction for MS patient CSF cells in MS case/control dataset. (A) UMAP visualization of MS patient CSF cells across batch correction strategies. Spliced and unspliced modalities were combined via concatenation or sum prior to correction with mutual nearest neighbors (MNN), ComBat, or Scanorama. Method performance was measured by batch label mixing metrics kBET and LISI (B), as well as the preservation of the relationship between spliced and unspliced counts (C). Distributions represent the per gene Pearson correlation between cell-cell distances in the phase space (unspliced, spliced) of corrected data and the cell-cell distances in the phase space of each individual MS patient. Dashed line represents the mean correlation.

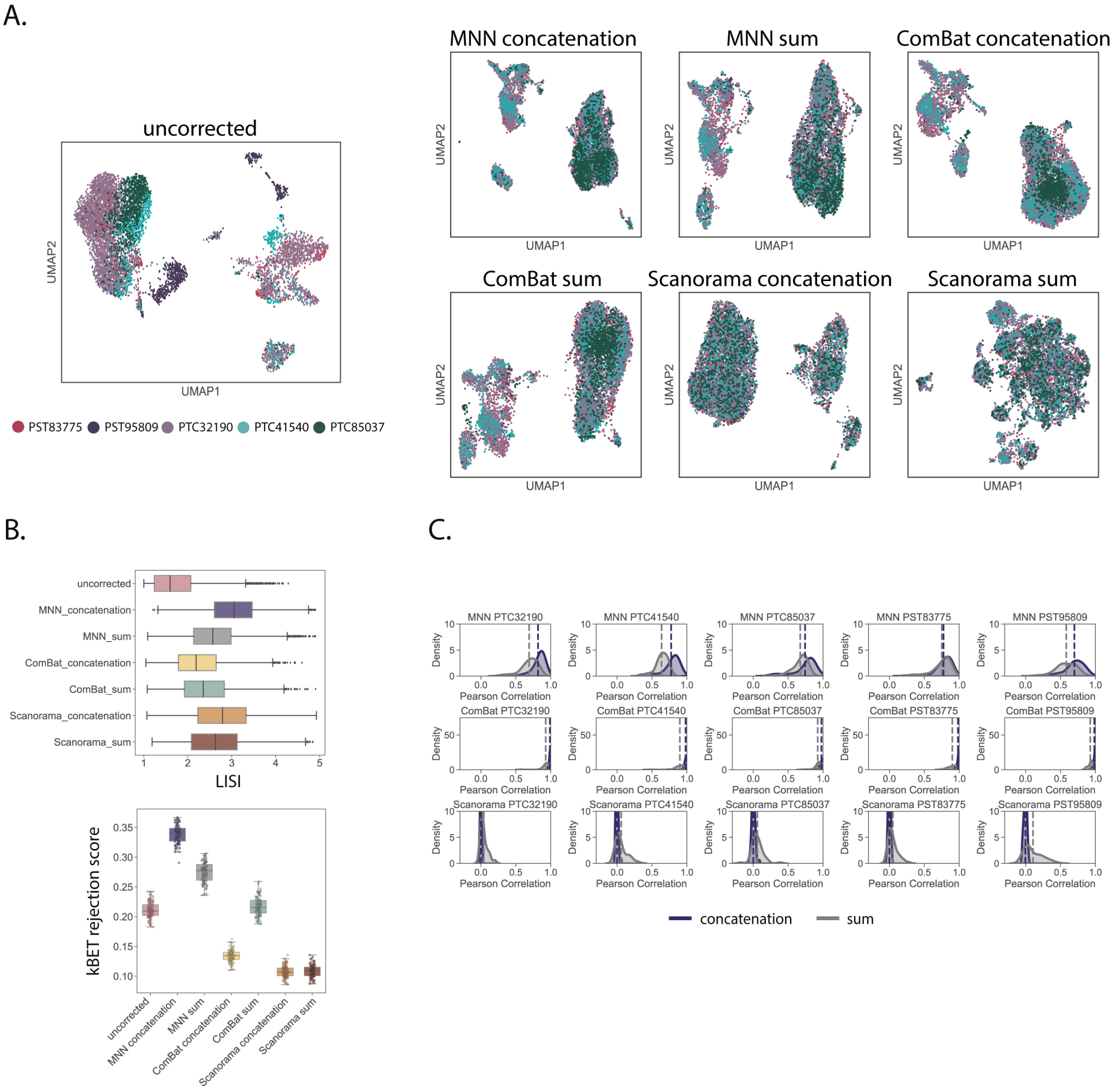

Figure S7: Evaluating batch effect correction for control patient CSF cells in MS case/control dataset. (A) UMAP visualization of control patient CSF cells across batch correction strategies. Spliced and unspliced modalities were combined via concatenation or sum prior to correction with mutual nearest neighbors (MNN), ComBat, or Scanorama. Method performance was measured by batch label mixing metrics kBET and LISI (B), as well as the preservation of the relationship between spliced and unspliced counts (C). Distributions represent the per gene Pearson correlation between cell-cell distances in the phase space (unspliced, spliced) of corrected data and the cell-cell distances in the phase space of each individual control patient. Dashed line represents the mean correlation.

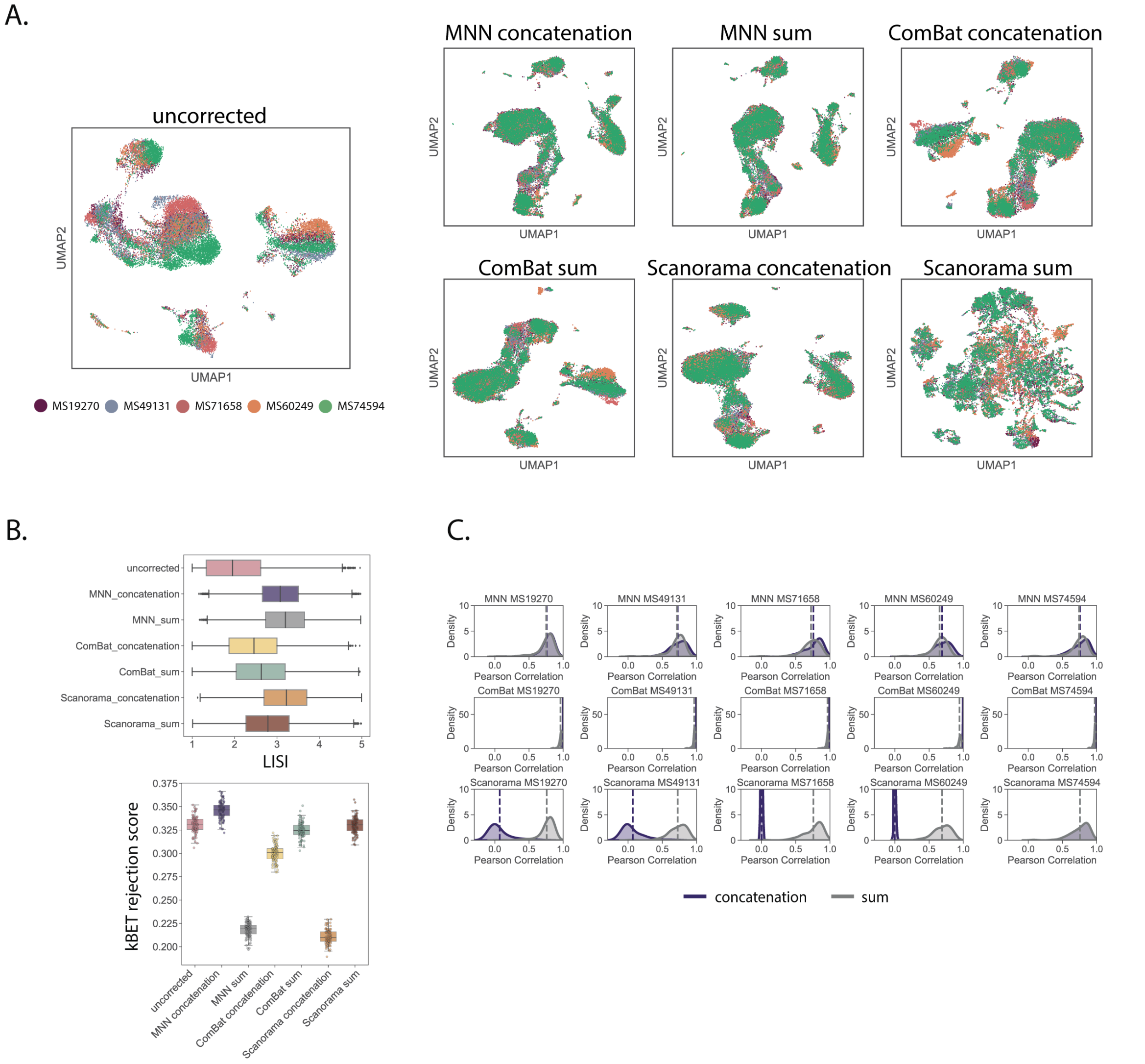

Figure S8: Evaluating batch effect correction for MS patient PBMCs in MS case/control dataset. (A) UMAP visualization of MS patient PBMCs across batch correction strategies. Spliced and unspliced modalities were combined via concatenation or sum prior to correction with mutual nearest neighbors (MNN), ComBat, or Scanorama. Method performance was measured by batch label mixing metrics kBET and LISI (B), as well as the preservation of the relationship between spliced and unspliced counts (C). Distributions represent the per gene Pearson correlation between cell-cell distances in the phase space (unspliced, spliced) of corrected data and the cell-cell distances in the phase space of each individual MS patient. Dashed line represents the mean correlation.

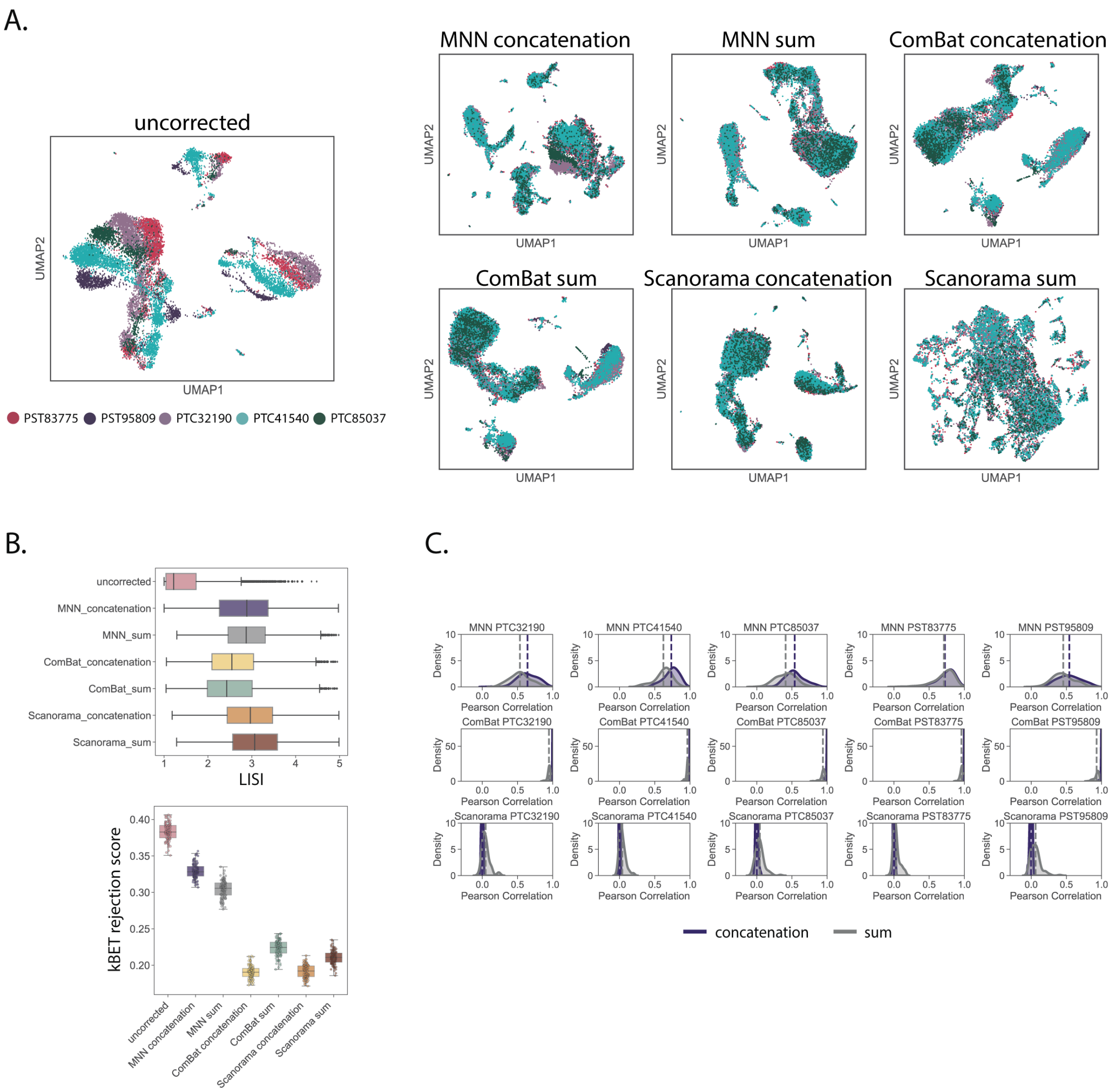

Figure S9: Evaluating batch effect correction for control patient PBMCs in MS case/control dataset. (A) UMAP visualization of control patient PBMCs across batch correction strategies. Spliced and unspliced modalities were combined via concatenation or sum prior to correction with mutual nearest neighbors (MNN), ComBat, or Scanorama. Method performance was measured by batch label mixing metrics kBET and LISI (B), as well as the preservation of the relationship between spliced and unspliced counts (C). Distributions represent the per gene Pearson correlation between cell-cell distances in the phase space (unspliced, spliced) of corrected data and the cell-cell distances in the phase space of each individual control patient. Dashed line represents the mean correlation.

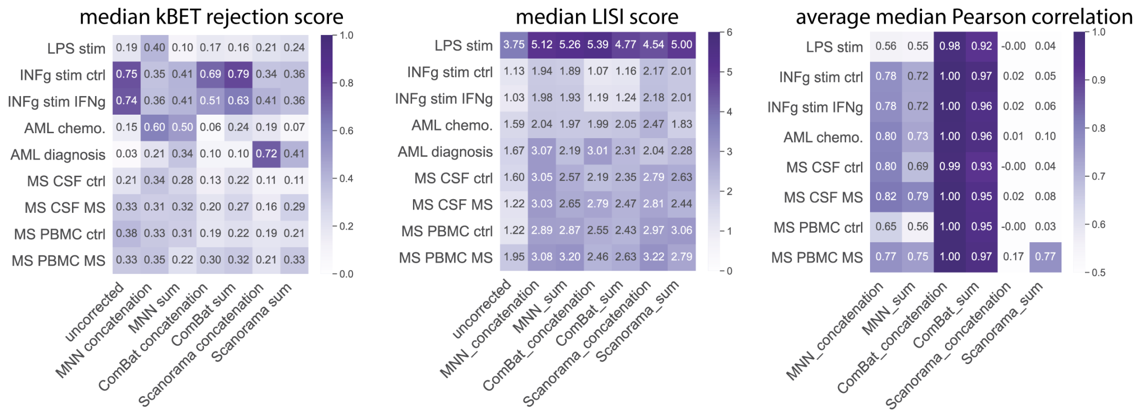

Figure S10: Overall performance of batch correction approaches across perturbation and disease datasets. Batch effect correction performance was assessed according to three metrics, including the median kBET rejection score, median LISI score, and average median Pearson correlation of phase space distances. A correction approach was selected for each dataset if it had the lowest kBET score, highest LISI score, and highest Pearson correlation score.

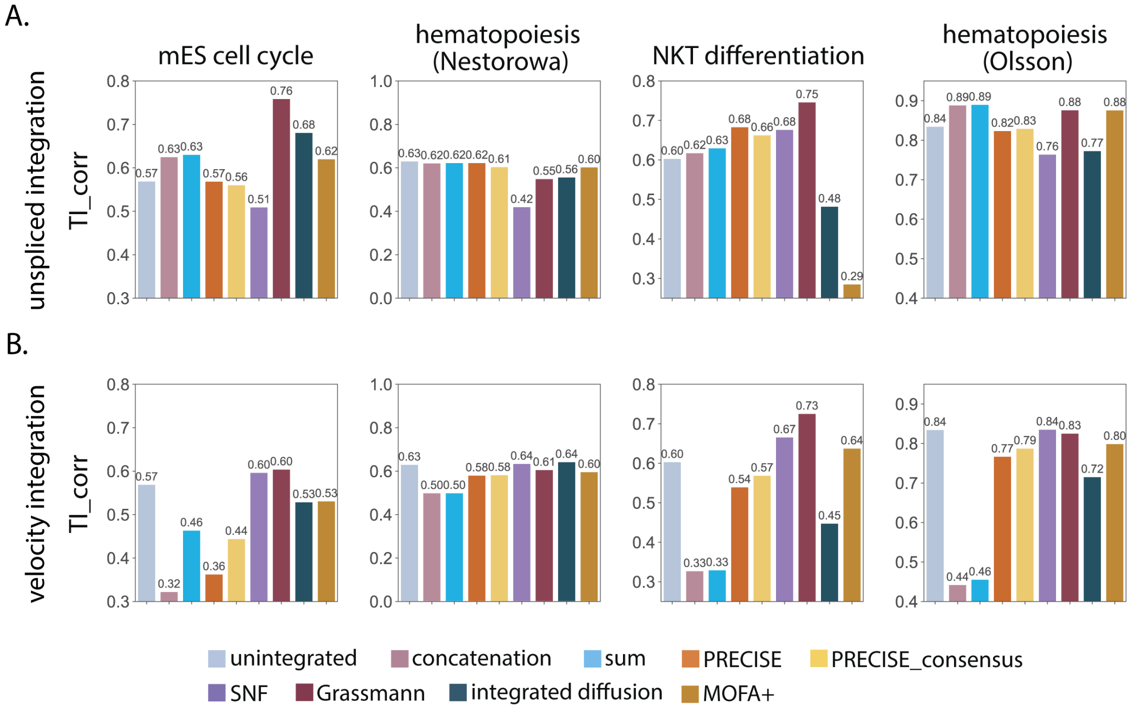

Figure S11: Integration method performance on inferring biological trajectories using Slingshot. Trajectory inference with Slingshot was performed to assess the quality of inferred embryonic cell cycle, hematopoiesis differentiation (Nestorowa), NKT cell differentiation, and hematopoiesis differentiation (Olsson) trajectories from (A) spliced and unspliced or (B) moments of spliced and RNA velocity integrated features generated from eight integration methods that infer a joint latent space. The barplots represent the trajectory inference correlation scores (TI_{corr}).

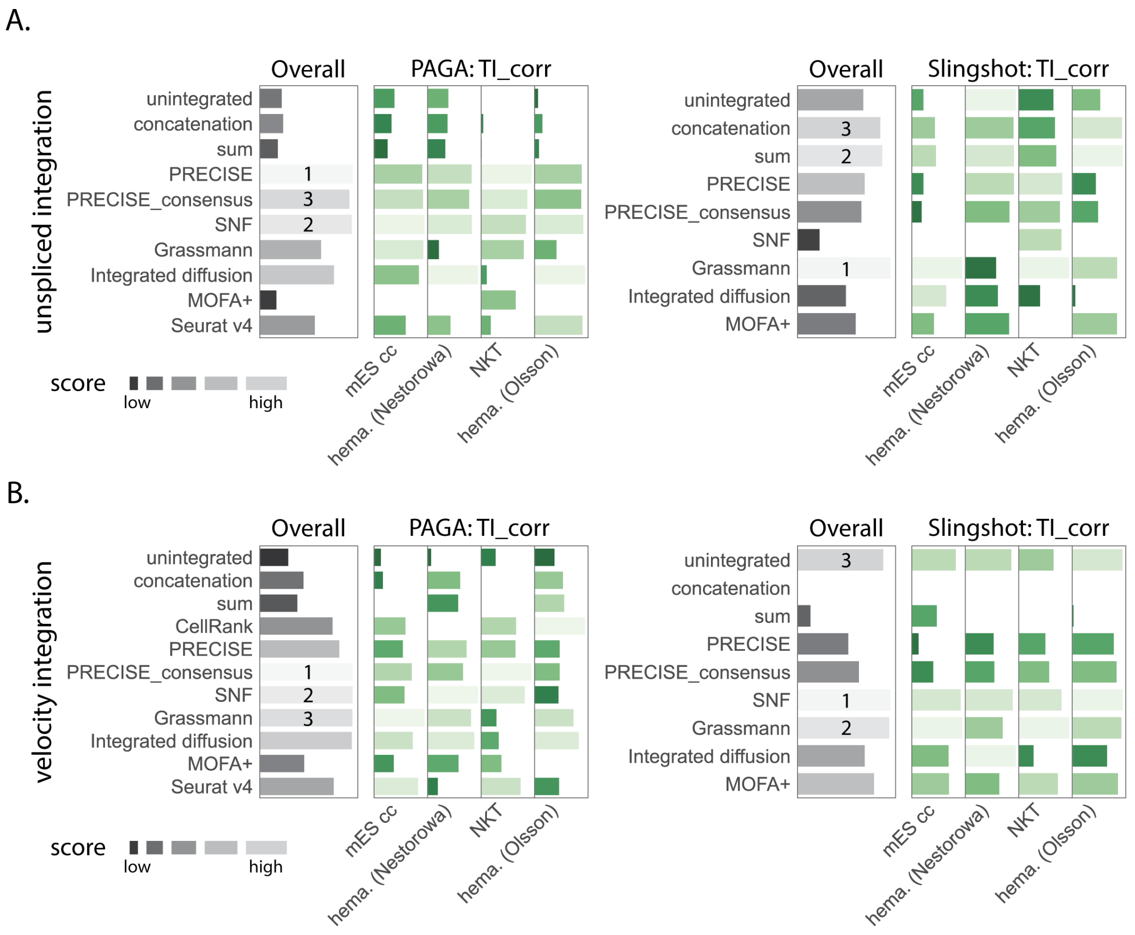

Figure S12: Ranked integration method performance for trajectory inference. Integration methods were ranked by their performance on inferring biological trajectories across mouse embryonic stem cell cycle (mES cc), mouse hematopoiesis (hema. (Nestorowa)), NKT cell differentiation, and mouse hematopoiesis (hema. (Olsson)) datasets. Individual methods were first ranked according to a trajectory inference correlation (TI_{corr}) score, which measures the harmonic mean of cellular positioning correlation and feature importance score correlation to a ground truth reference. The overall performance was then assessed by taking the average of ranked scores across datasets. (A) Overall quality of spliced and unspliced integration performance on inferring biological trajectories using the integrated graph and PAGA/diffusion pseudotime or the integrated embedding and Slingshot. (B) Overall quality of moments of spliced and RNA velocity integration performance on inferring biological trajectories using the integrated graph and PAGA/diffusion pseudotime or the integrated embedding and Slingshot. Here, a higher score is represented by a longer lighter bar. Across both datasets and modalities, intermediate integration approaches often outperform unintegrated data on trajectory inference.

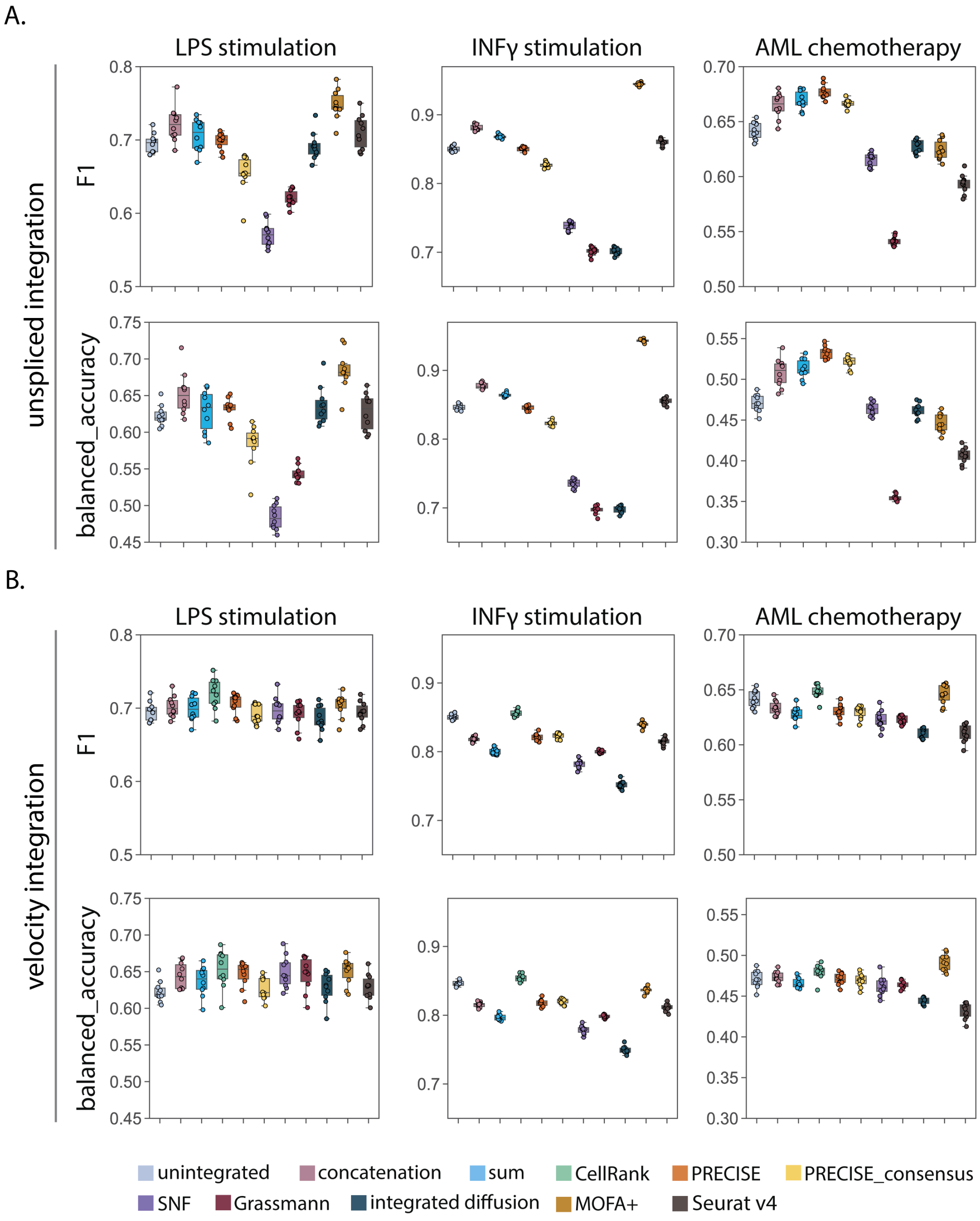

Figure S13: Integration performance on classifying cells according to perturbation condition labels using label propagation. Label propagation was used to classify cells according to treatment condition from (A) spliced and unspliced or (B) moments of spliced and RNA velocity integrated features generated from ten integration approaches. The boxplots represent classification accuracy according to two metrics, top panel: F1 score, bottom panel: balanced accuracy score.

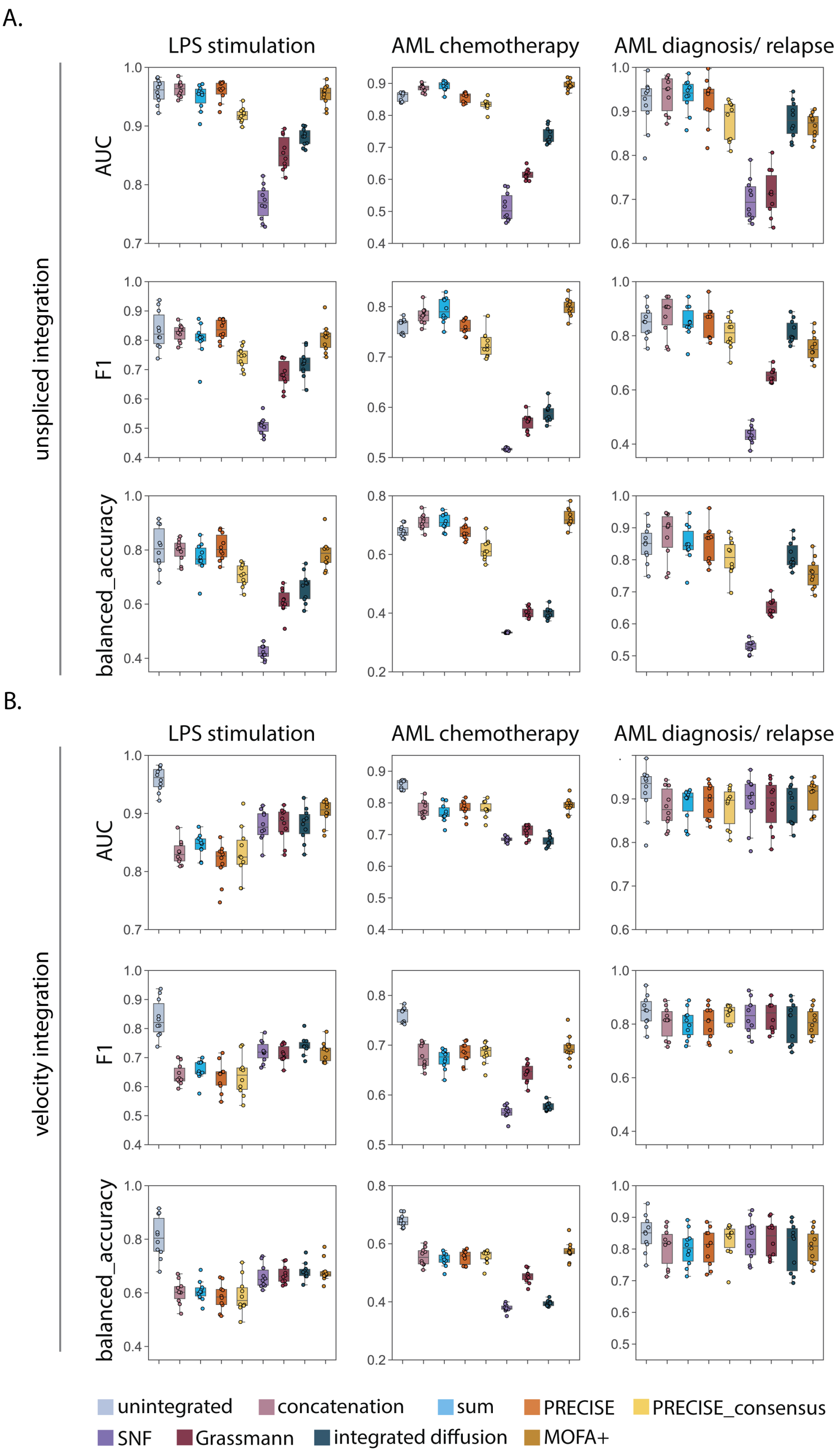

Figure S14: Integration performance on perturbation or disease status classification using a SVM classifier. A support vector machine (SVM) classifier was used to classify cells according to treatment condition or disease status from (A) spliced and unspliced or (B) moments of spliced and RNA velocity integrated features generated from eight integration approaches. The boxplots represent classification accuracy according to three metrics, including area under the receiver operator curve (AUC), F1 score, and balanced accuracy.

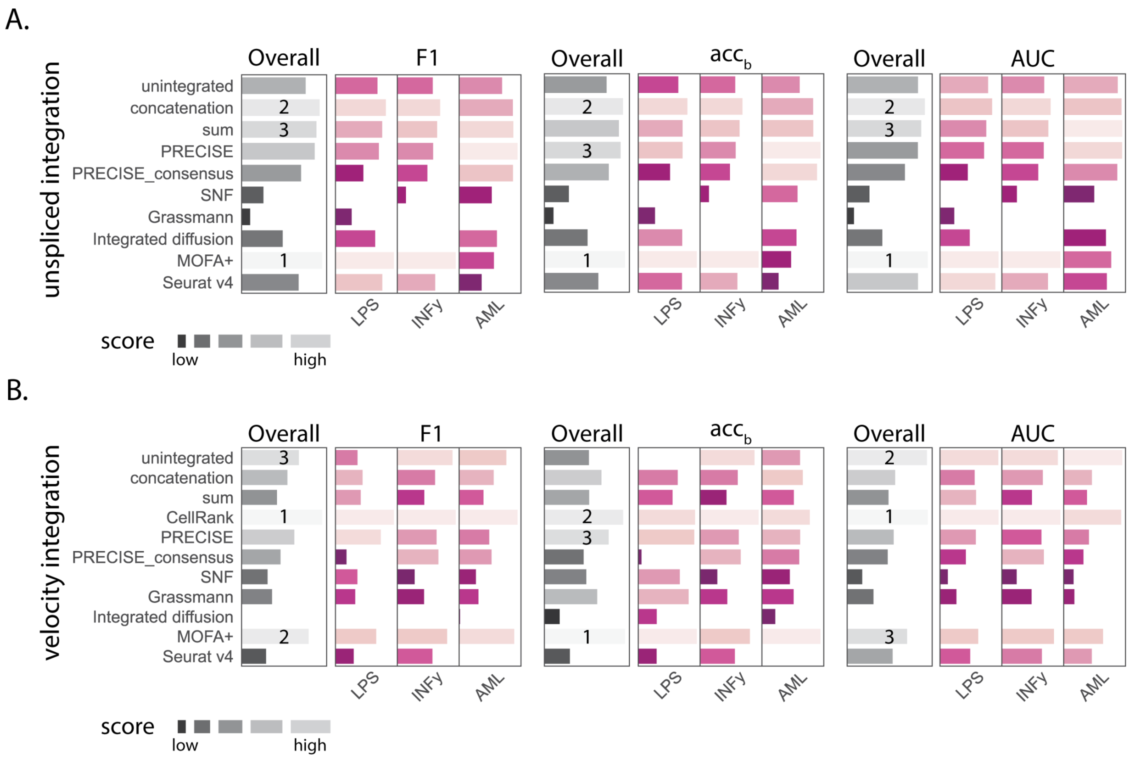

Figure S15: Ranked integration method performance for perturbation classification. Integration methods were ranked according to their performance on classifying cells according to perturbation condition across three datasets, including LPS stimulation of macrophage-like cells (LPS), INFγ stimulation of pancreatic islet cells (INFγ), and chemotherapy treated cells from a patient with Acute Myeloid Leukemia (AML). Label propagation was used to classify cells according to treatment condition and methods were evaluated by computing three metrics of success: F1 score, balanced accuracy (acc_b), and area under the receiver operator curve (AUC). The overall performance was then assessed by taking the average of ranked scores across datasets for each metric. (A) Overall quality of spliced and unspliced integration performance on classification of treatment condition. (B) Overall quality of moments of spliced and RNA velocity integration performance on classification of treatment condition. Here, a higher score is represented by a longer lighter bar. Across all three datasets and metrics, spliced and unspliced integration with MOFA+, concatenation, and sum outperformed unintegrated data on perturbation classification.

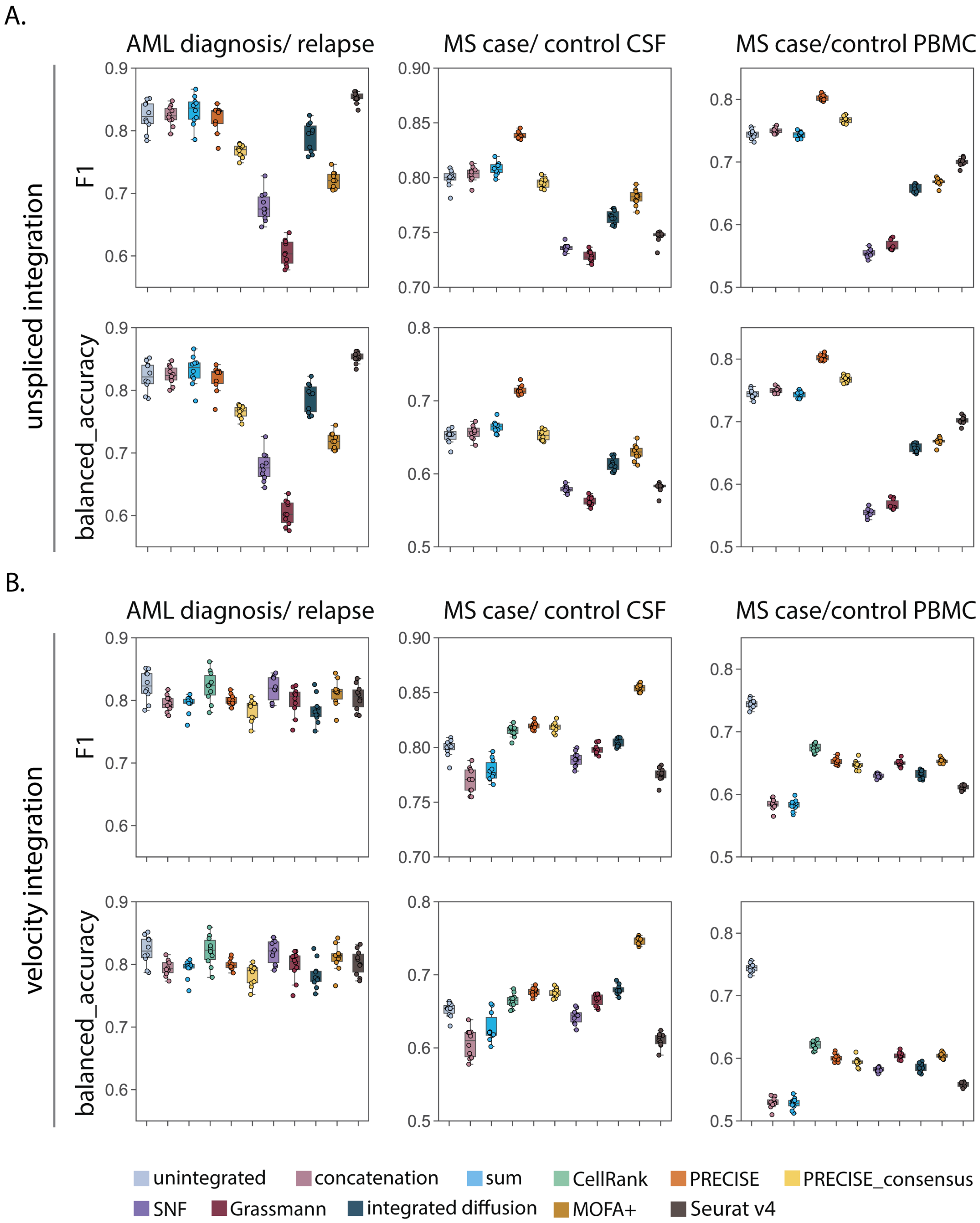

Figure S16: Integration performance on classifying cells according to patient disease status using label propagation. Label propagation was used to classify cells according to patient disease status from (A) spliced and unspliced or (B) moments of spliced and RNA velocity integrated features generated from ten integration approaches. The boxplots represent classification accuracy according to two metrics, top panel: F1 score, bottom panel: balanced accuracy.

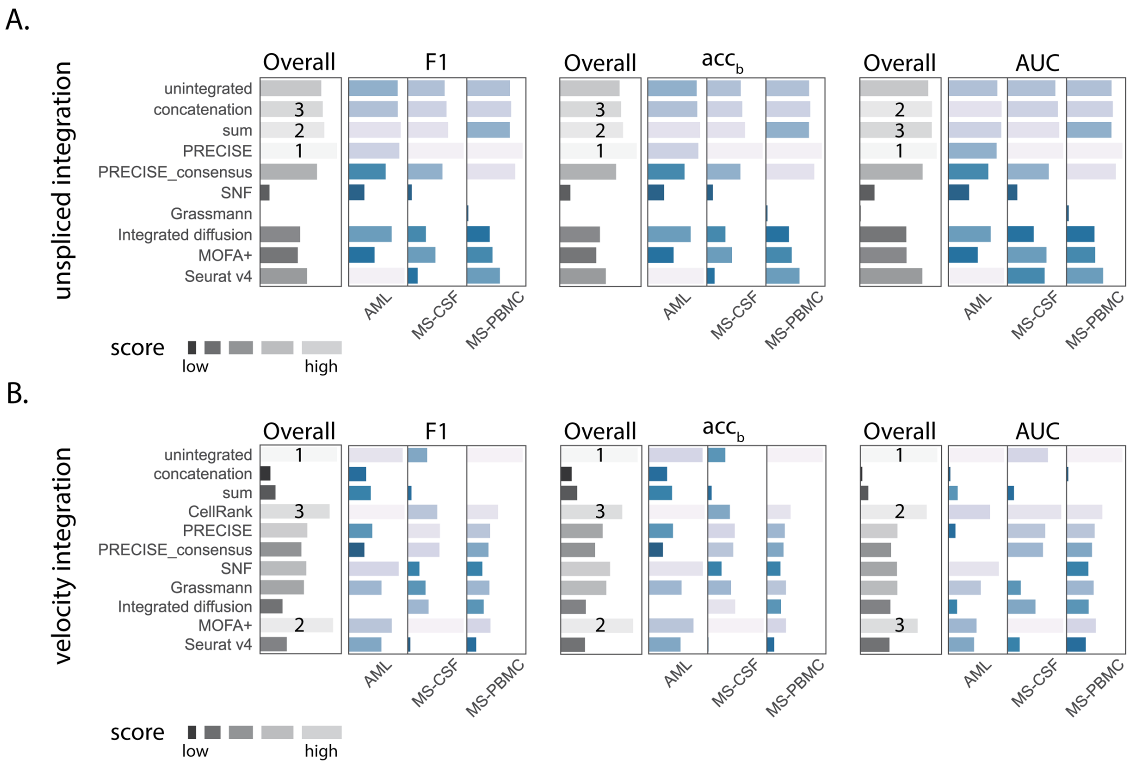

Figure S17: Ranked integration method performance on disease state classification. Integration methods were ranked according to their performance on predicting whether cells were from a healthy or disease patient across three datasets, including an Acute Myeloid Leukemia diagnosis and relapse dataset (AML), a Multiple Sclerosis case/control dataset of cerebral spinal fluid (MS-CSF), and a Multiple Sclerosis case/control dataset of peripheral blood mononuclear cells (MS-PBMC). Label propagation was used to classify cells according to patient disease status and methods were evaluated by computing three metrics of success: F1 score, balanced accuracy (acc_b), and area under the receiver operator curve (AUC). The overall performance was then assessed by taking the average of ranked scores across datasets for each metric. (A) Overall quality of spliced and unspliced integration performance on classification of cells according to patient disease status. (B) Overall quality of moments of spliced and RNA velocity integration performance on classification of cells according to patient disease status. Here, a higher score is represented by a longer lighter bar. Across all three datasets and metrics, spliced and unspliced integration with PRECISE, concatenation and sum outperformed unintegrated data on disease state prediction.
