# Supplementary material for: Integrating temporal single-cell gene expression modalities for trajectory inference and disease prediction
Source: Genome Biol. 2022 Sep 5;23:186. doi: 10.1186/s13059-022-02749-0 (PMC9442962; doi:10.1186/s13059-022-02749-0)
Supplement: Supplementary file 2 — Additional file 2. Review history. [file 13059_2022_2749_MOESM2_ESM.docx]

Review History

**First round of review**

**Reviewer 1**

**Were you able to assess all statistics in the manuscript, including the appropriateness of statistical tests used?**

Yes

**Comments to author:**

The paper describes a benchmarking study on methods for integrating temporal single-cell gene expression modalities. The integrated analysis of gene expression modalities is crucial for the interpretation of dynamic phenotypes. The authors addressed a computationally urgent task and should be of general interest to the single-cell community. Specifically, the authors benchmarked eight data integration methods and investigated integration of unspliced, spliced, and RNA velocity gene expression modalities for resolving discrete and continuous variation in cell and disease states. This paper concluded with some practical recommendations for task-specific integration of single-cell gene expression modalities. Overall, this is an interesting work, aiming to address a critical problem in temporal inference and data-driven approach in single-cell biology.

Major comments:

(1) The Method section was comprehensive and helpful to readers. However, it could be further improved if some detailed information that related to the methods such as"Concatenation" and "Sum" could be provided. Meanwhile, it would be helpful to provide some literature references for the two methods.

(2) The current integrating methods selected in this study are all data-driven approaches. The underlying assumptions for these methods during the integration should state clearly. Besides, are there any model based methods for integrating temporal single-cell gene expression modalities?

(3) Computational cost is a huge consideration in the emerging large-scale single-cell expression data integrations. It would be interesting if the authors could address the scalability issues of the integrative methods.

Minor comments:

The authors evaluated the methods on how well integrated features infer biological trajectories classify a cell's response to a drug perturbation, or classify the disease status of a cell based on a prior knowledge or annotations. It would be interesting if any new biological insights that could be gained through the integrative study.

**Reviewer 2**

**Were you able to assess all statistics in the manuscript, including the appropriateness of statistical tests used?**

Yes

**Comments to author:**

Summary of the article: Most existing trajectory inference algorithms except Velocity infer trajectory based on gene expression similarity and cannot determine the direction of trajectories. Integrating gene expression matrix with velocity outputs can overcome this limitation. Several methods such as CellRank have adopted this strategy. Ranek et al. evaluated if integrating velocity outputs can improve trajectory inference and benchmarked different integration methods. Similarly, the authors also evaluated if the integration can aid in predicting cell states.

Major concerns:

1. In addition to the evaluated integration methods, other multi-modality integration methods such as Seurat v4 and MOFA+ can be also benchmarked as these two methods perform well for single-cell multi-modality integration tasks.

2. Ranek et al. performed trajectory inference using Partition-based Graph Abstraction followed by diffusion pseudotime as this approach was shown to outperform other methods for inference of complex differentiation topologies. It is recommended to try some other methods especially from other categories such as tree methods, cyclic methods, etc. The performance of trajectory inference could be data-dependent. It is unlikely to have one method that fit all datasets.

3. Ranek et al. used ComBat and MNN for batch correction of sliced and unspliced counts. However, ComBat does not perform well in batch correction according to existing benchmarking studies. Besides ComBat and MNN, there are some other methods that also output batched corrected values such as Seurat, Scanaroma, etc according to Figure 5 of https://www.nature.com/articles/s41592-021-01336-8 (article title: Benchmarking atlas-level data integration in single-cell genomics, by Theis' lab)

4. Ranek et al. evaluated inference of biological trajectories using only two single-cell RNA sequencing datasets representing the cell cycle and stem cell differentiation. More datasets should be included for assessment of trajectory inference, as it is the major component of the presented benchmark study.

**Authors Response**

**Point-by-point responses to the reviewers’ comments:**

The paper describes a benchmarking study on methods for integrating temporal single-cell gene

expression modalities. The integrated analysis of gene expression modalities is crucial for the

interpretation of dynamic phenotypes. The authors addressed a computationally urgent task and

should be of general interest to the single-cell community. Specifically, the authors benchmarked

eight data integration methods and investigated integration of unspliced, spliced, and RNA

velocity gene expression modalities for resolving discrete and continuous variation in cell and

disease states. This paper concluded with some practical recommendations for task-specific

integration of single-cell gene expression modalities. Overall, this is an interesting work, aiming

to address a critical problem in temporal inference and data-driven approach in single-cell

biology.

Major comments:

1. The Method section was comprehensive and helpful to readers. However, it could be

further improved if some detailed information that related to the methods such as

"Concatenation" and "Sum" could be provided. Meanwhile, it would be helpful to

provide some literature references for the two methods.

*Based upon the reviewer’s suggestion, we have now provided additional notation and*

*literature references for concatenation and sum in the section ‘Selection of Integration*

*Methods’ in the main text (lines 173-175) and ‘Integration Methods’ in the methods*

*section (lines 710, 714-715).*

2. The current integrating methods selected in this study are all data-driven approaches. The

underlying assumptions for these methods during the integration should state clearly.

Besides, are there any model based methods for integrating temporal single-cell gene

expression modalities?

*We thank the reviewer for this comment and suggestion. We have further elaborated on*

*any underlying assumptions for the integration methods evaluated in this study in the*

*‘Integration Methods’ section in the methods (e.g. CellRank lines 721-724, SNF lines*

*753-755, Integrated diffusion lines 810-811, MOFA+ lines 824-828). Furthermore, we*

*have identified one model based multi-omics data integration method, MOFA+: (see*

*Argelaguet et al., PMID 32393329). Based upon all reviewers’ suggestions, we have now*

*evaluated MOFA+, as well as another commonly used data-driven method, Seurat v4 (see*

*Hao et al., PMID 34062119), on all prediction tasks, including trajectory inference, classification of perturbation states, and classification of disease cell states. For details on*

*MOFA+ implementation see lines 189-195 and 819-829. For details on Seurat v4*

*implementation see lines 182-188 and 830-842.*

3. Computational cost is a huge consideration in the emerging large-scale single-cell expression data integrations. It would be interesting if the authors could address the scalability issues of the integrative methods.

*We thank the reviewer for this suggestion and completely agree that computational cost is an important practical consideration when selecting an integration strategy. We have now provided a scalability analysis to further highlight this by increasing the amount of cells or features prior to performing integration (see scalability analysis in the methods section lines 945-958 for more details). By measuring the total elapsed time, we found the simple integration strategies (e.g. sum, concatenation and PRECISE) to be the most efficient when varying the number of cells or features, whereas SNF, MOFA+ and integrated diffusion required increasing amounts of compute time. The results of these analyses are shown in Figure 6 (lines 432-442).*

Minor comments:

1. The authors evaluated the methods on how well integrated features infer biological trajectories classify a cell's response to a drug perturbation, or classify the disease status of a cell based on a prior knowledge or annotations. It would be interesting if any new biological insights that could be gained through the integrative study.

*We would like to thank the reviewer for this comment. While we agree with the reviewer that it would be interesting to see if any new biological insights could be gained from an integrative study of temporal gene expression modalities, we aimed the focus of this work to provide users with a framework for how to select a method and a set of modalities based upon the task of interest. We have now acknowledged this comment as an important area for future study in the ‘Discussion’ section of the main text (lines 479-489) and have described additional computational approaches that may be leveraged on an integrated dataset to evaluate this further.*

**Reviewer #2:**

Most existing trajectory inference algorithms except Velocity infer trajectory based on gene expression similarity and cannot determine the direction of trajectories. Integrating gene expression matrix with velocity outputs can overcome this limitation. Several methods such as CellRank have adopted this strategy. Ranek et al. evaluated if integrating velocity outputs can improve trajectory inference and benchmarked different integration methods. Similarly, the authors also evaluated if the integration can aid in predicting cell states.

Major comments:

1. In addition to the evaluated integration methods, other multi-modality integration methods such as Seurat v4 and MOFA+ can be also benchmarked as these two methods perform well for single-cell multi-modality integration tasks.

*We thank the reviewer for these data integration method suggestions and have now evaluated Seurat v4 and MOFA+ on all prediction tasks, including trajectory inference, classification of perturbation states, and classification of disease cell states. Similar to our previous results, we found unspliced and RNA velocity integration with Seurat v4 and MOFA+ led to improved trajectory inference performance when compared to unintegrated data (as shown in Figure 2). Moreover, we found MOFA+ to be the top performing integration method for leveraging unspliced counts to infer perturbation states (as shown in Figure 5A). For details on MOFA+ implementation see lines 189-195 and 819-829. For details on Seurat v4 implementation see lines 182-188 and 830-842.*

2. Ranek et al. performed trajectory inference using Partition-based Graph Abstraction followed by diffusion pseudotime as this approach was shown to outperform other methods for inference of complex differentiation topologies. It is recommended to try some other methods especially from other categories such as tree methods, cyclic methods, etc. The performance of trajectory inference could be data-dependent. It is unlikely to have one method that fit all datasets.

*Based upon the reviewer’s recommendation, we have additionally evaluated Slingshot (Street et al., PMID 29914354), a top performing trajectory inference method for inferring tree-based differentiation topologies (see lines 853-870 for implementation). For results of this analysis, please see ‘Integration performance on inference of biological trajectories’ section in the main text (lines 332-342) and Supplementary Figures 11 and 12. Trajectory inference performance certainly varies based upon the how well the trajectory inference method fits the underlying differentiation trajectory; however, we find integrated data still outperforms unintegrated data across inference methods for some datasets (e.g. unspliced and spliced integration from mouse embryonic cell cycle, hematopoiesis (Olsson), and NKT cell differentiation).*

*Furthermore, we tested integration performance using a cyclical trajectory inference method, angle, on the cell cycle dataset (see ‘angle’ in ‘Off-the-shelf’ section in the methods section of Saelens et al., PMID 30936559). However, we observed poor predictive performance across all integration strategies (< 0.45) in comparison to PAGA (Figure 2) or slingshot (Supplementary Figure 11).*

3. Ranek et al. used ComBat and MNN for batch correction of sliced and unspliced counts. However, ComBat does not perform well in batch correction according to existing benchmarking studies. Besides ComBat and MNN, there are some other methods that also output batched corrected values such as Seurat, Scanaroma, etc according to Figure 5 of https://www.nature.com/articles/s41592-021-01336-8 (article title: Benchmarking atlas-level data integration in single-cell genomics, by Theis' lab)

*We would like to thank the reviewer for this recommendation and literature reference. We have now evaluated Scanorama in addition to MNN and ComBat, as all three provide corrected counts and are shown to be the best performers for recovering cell states according to Figure 5 of the atlas-level data integration benchmarking study by Theis’ lab, PMID 34949812. The updated batch effect correction results are shown in Supplementary Figures 1-10 and further explained in the ‘Batch effect correction’ section of the methods. As ComBat remains sufficient at removing batch effects in the datasets selected for this study (e.g. low kBET rejection score, high LISI score, high Pearson correlation of phase space pairwise distances as shown in Supplementary Figure 10), we have decided to continue to use it for most perturbation and disease datasets, as outlined in Supplementary Table 1.*

4. Ranek et al. evaluated inference of biological trajectories using only two single-cell RNA sequencing datasets representing the cell cycle and stem cell differentiation. More datasets should be included for assessment of trajectory inference, as it is the major component of the presented benchmark study.

*Based upon the reviewer’s suggestion, we have now evaluated trajectory inference performance using Slingshot and PAGA on four single-cell RNA sequencing datasets representing the cell cycle and stem cell differentiation. Across all four datasets and trajectory inference methods, we found integration with unspliced and spliced counts led to increased trajectory inference correlation scores, with the intermediate integration methods outperforming the simple strategies (as shown in Figure 2, Figure 5, and Supplementary Figure 12). In contrast, RNA velocity integration led to increased performance when using PAGA for inference (Figure 2), yet achieved a similar or modest increase in performance across datasets when using Slingshot (Supplementary Figures 11 and 12). For more details on the additional datasets selected, please see the ‘Selection of Datasets’ section in the main text (lines 60-91), ‘Datasets’ section in the methods (lines 534-547), and Supplementary Tables 1 and 3.*

**Second round of review**

**Reviewer 1**

**Comments to author:**

All my concerns have been addressed.
